# Characterization of *Cystoisospora suis* sexual stages *in vitro*

**DOI:** 10.1186/s13071-020-04014-4

**Published:** 2020-03-18

**Authors:** Anna Sophia Feix, Teresa Cruz-Bustos, Bärbel Ruttkowski, Anja Joachim

**Affiliations:** Institute for Parasitology, Department of Pathobiology, University of Veterinary Medicine Vienna, Veterinaerplatz 1, Vienna, 1210 Austria

**Keywords:** *Isospora suis*, Gametes, *DLC-1*, *HAP2*, *OWP*, *RAD51/Dmc*, *Nima*-related protein kinases

## Abstract

**Background:**

The porcine coccidium *Cystoisospora suis* is characterized by a complex life-cycle during which asexual multiplication is followed by sexual development with two morphologically distinct cell types, the micro- and macrogametes. Genes related to the sexual stages and cell cycle progression were previously identified in related Apicomplexa. *Dynein light chain type 1* and male gamete fusion factor *HAP2* are restricted to microgametes. Tyrosine-rich proteins and oocyst wall proteins are a part of the oocyst wall. The Rad51/Dmc1-like protein and Nima-related protein kinases are associated with the cell cycle and fertilization process. Here, the sexual stages of *C. suis* were characterized *in vitro* morphologically and for temporal expression changes of the mentioned genes to gain insight into this poorly known phase of coccidian development.

**Methods:**

Sexual stages of *C. suis* developing *in vitro* in porcine intestinal epithelial cells were examined by light and electron microscopy. The transcriptional levels of genes related to merozoite multiplication and sexual development were evaluated by quantitative real-time PCR at different time points of cultivation. Transcription levels were compared for parasites in culture supernatants at 6–9 days of cultivation (doc) and intracellular parasites at 6–15 doc.

**Results:**

Sexual stage of *C. suis* was detected during 8–11 doc *in vitro*. Microgamonts (16.8 ± 0.9 µm) and macrogamonts (16.6 ± 1.1 µm) are very similar in shape and size. Microgametes had a round body (3.5 ± 0.5 µm) and two flagella (11.2 ± 0.5 µm). Macrogametes were spherical with a diameter of 12.1 ± 0.5 µm. Merozoite gene transcription peaked on 10 doc and then declined. Genes related to the sexual stages and cell cycle showed an upregulation with a peak on 13 doc, after which they declined.

**Conclusions:**

The present study linked gene expression changes to the detailed morphological description of *C. suis* sexual development *in vitro*, including fertilization, meiosis and oocyst formation in this unique model for coccidian parasites. Following this process at the cellular and molecular level will elucidate details on potential bottlenecks of *C. suis* development (applicable for coccidian parasites in general) which could be exploited as a novel target for control.
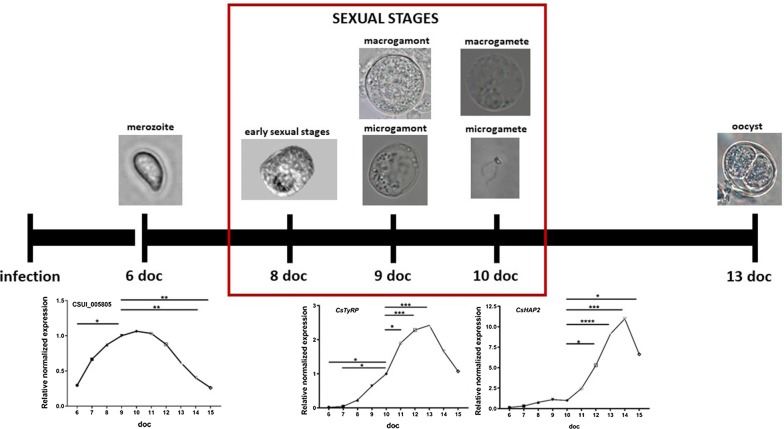

## Background

*Cystoisospora suis* (syn. *Isospora suis* Biester & Murray, 1934) is a coccidian parasite of swine which causes diarrhea and reduced weight gain in suckling piglets, mostly in the first three weeks of life, and leads to unthriftiness at weaning, considerably impairing animal health and productivity [1–4]. As with all coccidian parasites, the life-cycle of *C. suis* is characterized by asexual multiplication (sporogony, merogony) followed by sexual development with two morphologically distinct cell types, the micro- and macrogametes which presumably fuse to form a zygote from which the oocyst arises [5, 6].

Previous studies have shown that the development of *C. suis in vitro* is comparable with the life-cycle *in vivo*; however, the development of sexual stages *in vitro* is delayed [7]. This makes it possible to observe, harvest and examine sexual stages in the short time frame in which they occur. Macrogametes of coccidians form without division and are large and immobile, while microgamonts divide several times to form microgametes, consisting of a small body and flagella which are used to move quickly in search of macrogametes [8].

After fertilization by a microgamete the wall-forming bodies of the macrogamete fuse and form the oocyst wall, as seen in other Coccidia [9, 10]. In the coccidian parasite *Eimeria* two types of wall-forming bodies can be found, an electron dense form, giving rise to the outer layer of the oocyst, and sponge-like bodies that fuse to form the inner layer of the oocyst wall [9].

Transcriptomic and proteomic analyses carried out in *Eimeria* and *Toxoplasma* revealed different genes coding for proteins related to the sexual stages [11, 12]. The two main protein families participating in oocyst wall formation in coccidia are tyrosine-rich proteins called GAM [13] and cysteine-rich proteins OWPs, first described in detail in *Cryptosporidium* [14, 15]. GAM proteins are found in the wall-forming bodies type 1. Proteases break down GAM proteins into tyrosine-rich peptides, which are then oxidatively crosslinked by peroxidases and incorporated into the oocyst wall in dityrosine bonds [16, 17]. GAM proteins are the most studied proteins of the oocyst wall; they were developed as antigens for transmission blocking vaccines targeting the gametocyte-specific proteins GAM56, GAM82 and GAM22 of *Eimeria* [13, 18–20]. Cysteine-rich OWPs have also been described in *Toxoplasma gondii* [21]. They are found in the wall-forming bodies type 2. The cysteine residues form disulfide bridges responsible for the stabilization and formation of the oocyst and sporocyst walls, conferring additional strength and rigidity [22]. Proteins with important roles in apicomplexan male sexual stages are involved in axoneme and flagella assembly and construction [23], DNA replication [24], microgamete budding from microgamonts [25] and gamete fusion [26]. Upregulated expression of genes coding for tubulins, dyneins, radial spokes, basal body family proteins, a certain family protein, kinesins, enkurin-related protein, HAP2 and intraflagellar transport proteins was observed by RNA Seq analysis of *Eimeria tenella* sexual stages [27]. HAP2 is essential for the fusion of gametes surface membrane and subsequent fertilization [27], and it has been proposed as a possible candidate for a transmission blocking vaccine in apicomplexan parasites [28, 29].

The process of fertilization in Coccidia is still poorly understood and has not been visualized yet, but it is commonly assumed that, that following fusion of the micro- and macrogamete, the apicomplexan zygote develops into an unsporulated oocyst. The oocysts are excreted to the environment and the development continues, and meiosis and mitosis result in infectious haploid sporozoites [17, 30]. Among the genes thought to be involved in these processes are the meiotic recombination *Rad51* and *Dmc1*, *protein kinase*, *Aurora* and *Nima* genes, C*yclin dependent kinase* and *Polo* genes [15, 31, 32].

In this study we aimed to provide a first characterization of *C. suis* sexual stages *in vitro* by comparing the morphology of the stages and the transcriptional profiles of selected conserved genes related to the sexual development of apicomplexan parasites. We hypothesized (i) that all developmental stages of *C. suis* occur *in vitro* and are comparable to those *in vivo* experiments, although the *in vitro* development of the parasite takes longer compared to infections of piglets; and (ii) that during sexual development, transcription of genes related to this life-cycle phase is increased in *C. suis* similar to other Coccidia and *Plasmodium*.

## Methods

### *Cystoisospora suis* oocyst collection

*Cystoisospora suis* oocysts (strain Wien 1) were obtain from experimental infected suckling piglets. Piglets were raised with the sow in the animal facilities of the Institute of Parasitology, University of Veterinary Medicine Vienna, Austria. Infection of piglets, oocyst collection, oocyst isolation, oocyst purification and excystation were performed as described previously [7]. In deviation to the original protocol, after NaOCl treatment and washing, the oocysts were vortexed three times for 45 s with Precellys® glass beads (Peqlab, Erlangen, Germany) in 2% sodium taurocholate hydrate (Sigma-Aldrich, St. Louis, USA) in DMEM/Ham’s F-12 culture medium (Gibco, Thermo Fisher Scientific, Waltham, USA).

### *In vitro* culture and parasite harvest

Intestinal porcine epithelial cells (IPEC-1, ACC 705, Leibniz Institute DSMZ-German Collection of Microorganisms and Cell Cultures GmbH, Leibniz, Germany) [33–35] were used as host cells *in vitro* (seeded 4 × 10^5^ per well in a 6-well plate), in a DMEM/Ham’s F-12 medium (Gibco) with 5% fetal calf serum (FCS, Gibco) and 100 U/ml penicillin and 0.1 mg/ml streptomycin (PAA, Pasching, Austria) at 37 °C in 5% CO_2_. After 24 h of cell growth IPEC-1 were infected with 5 × 10^3^ sporozoites released from excysted oocysts and incubated further at 40 °C. Free sexual stages were harvested by collecting culture medium supernatant daily at 9–11 days of cultivation (doc). The collected stages were washed with phosphate-buffered saline (PBS; Gibco; 144.0 mg/l KH_2_PO_4_, 9000.0 mg/l NaCl, 795.0 mg/l Na_2_HPO_4_-7H_2_O) and purified using a Percoll® (GE Healthcare, Uppsala, Sweden) density gradient with layers at 80%, 40% and 30%, and the sample on top. The gradient was centrifuged at 600×*g* for 10 min at 20 °C in a Mega Star 3.0R swing bucket centrifuge (VWR International, Leuven, Belgium). Both acceleration and deceleration were at the lowest possible setting. For the sampling of intracellular sexual stages, adhering host cells and parasites were incubated with Accutase® (Thermo Fisher Scientific, Waltham, USA) for 30 min and the detached material was washed twice with PBS and pelleted by centrifugation at 600×*g* for 10 min. The numbers of sexual stages were estimated in the cell culture chambers and counted in a Neubauer-counting chamber at each given time point, and the mean numbers of sexual stages per well were calculated.

### Light and electron microscopy

Digital microphotographs of all *in vitro C. suis* stages, but especially isolated sexual stages, both live and fixed in 100% EtOH, were taken with an Olympus IX71 inverse microscope (Olympus, Shinjuku, Japan) or a Zeiss Imager Z2 microscope (Zeiss, Jena, Germany) at 400× and 600× magnification and measured (*n* = 50 per stage) with Zeiss ZEN lite software (Zeiss).

For scanning electron microscopy sample preparation coverslips were washed in 100% EtOH and coated with 0.1% poly-d-lysine (Merck Millipore, Burlington, USA) on which the isolated sexual stages were left to settle for 1 h at 36 °C in PBS. Afterwards the parasites were fixed for 3 min on the cover slip using 2.5% glutaraldehyde in PBS. The samples were washed twice in PBS for 15 min. Post-fixation was performed with 1% osmium tetroxide for 3 min. The coverslips were dehydrated in an ascending alcohol series from 30–100% ethanol for 3 min each. Thereafter the samples were critical point dried in a Leica CPD 300 (Leica Microsystems, Wetzlar, Deutschland). The dried samples were mounted on metal stubs and gold sputtered for 80 s with a JEOL JFC-2300HR (JEOL GmbH, Freising, Germany). All scanning electron microscopy work was performed at the Core Facility Cell Imaging and Ultrastructure Research, University of Vienna-member of the Vienna Life-Science Instruments (VLSI). Sexual stages were photographed with a JEOL IT 300 scanning electron microscope (JEOL) and measured with Zeiss ZEN lite software (Zeiss).

### Transcription levels at different time points of *C. suis* development *in vitro*

Quantitative real-time PCR (qPCR) was used to quantify the transcripts levels of four genes related to sexual stages, four genes related to cellular division and meiosis and one related to the merozoite stage of *C. suis* at different time points of development *in vitro*.

Total RNA was isolated from infected cell cultures using an RNeasy® mini kit (Qiagen, Hilden, Germany) and treated with RNase-free DNase (Qiagen) according to the manufacturer’s instructions to remove any DNA contamination. Total RNA was quantified using a NanoDrop® 2000 (Thermo Fischer Scientific, Waltham, MA, USA). cDNA synthesis was accomplished using the iScript cDNA synthesis kit (Bio-Rad, Hercules, California, USA).

The nucleotides sequences for genes linked with sexual development in Apicomplexa (Table 1) were searched using the Basic Search Alignment Tool (BLAST) in the genomic resource database ToxoDB (https://toxodb.org/toxo/). Alignment analysis and calculation of percentage identity were performed using Clustal omega (https://www.ebi.ac.uk/Tools/msa/clustalo).

**Table 1:** Primers used in this study

	Primer	Sequence (5′–3′)
1	Fw-ACTIN	CTTGCTGGCCGTGATTTGAC
2	Rv-ACTIN	ATATTGCCGTCCGGAAGCTC
3	Probe-ACTIN	CCTCCGCCGAGAAGGAAATT
4	Fw-GAPDH	TTCAACGAGAAGGAGCCAAG
5	Rv-GAPDH	CTTCGGAGGTGCAGACATG
6	Probe-GAPDH	CAAGGAAAAGGCTGAGGCGCAT
7	Fw-HAP2	GGAACCCAGGGAAATTTTGT
8	Rv-HAP2	CATGTTGTTGATGTGCGTGA
9	Probe-HAP2	GCTGCTGGTGCAGTGAGGTC
10	Fw-DLC1	TGCTATGGCCTGTTGATATGC
11	Rv-DLC1	CTTCTGGTCGAGCTCCTTTT
12	Probe-DLC1	TGCTGCGCGTGACTGTATAATCCA
13	Fw-OWP1	CCAGAAGGATGTTTATTTGCCG
14	Rv-OWP1	TGGGCAGATGTATTCAGGTTC
15	Probe-OWP1	AATCCGAAGGGCAGCGTTGTAGAA
16	Fw-TyRP	GAACTGGACGGTGATCGTGA
17	Rv-TyRP	GCTCTCAATAAGTCCCTCAGAG
18	Probe-TyRP	CTCATGCGCTCGCTACCTGA
19	Fw-Rad51	GCTTCGCTTTGCTTATTGTC
20	Rv-Rad51	CAACAACAGCCACACCATAC
21	Probe-Rad51	TGCCACGGCCCTATACAGGT
22	Fw-NIMA1	GCTGGAAACTGGTGTTTTAC
23	Rv-NIMA1	GCATCGCAGTACTCCATAAC
24	Probe-NIMA1	GTGAACTTCGGCACCCCAAC
25	Fw-NIMA2	AGGACAACTACATCCGTGTC
26	Rv-NIMA2	CGTGACATATATTTCGCTGA
27	Probe-NIMA2	TCCAGCAAGCAAGAACGCAG
28	Fw-NIMA4	AAAGAGTCGCAGATTCTCAG
29	Rv-NIMA4	CCTGCGTATGATCAAGAAGT
30	Probe-NIMA4	AGAATTCGCCTGGCGGATTT
31	Fw- CSUI_005805	CCTGAAAGTCGCCTGTCCAT
32	Rv- CSUI_005805	GACGCGTCAGCCGTTATAGT
33	Probe-CSUI_005805	CTCTCAGTTTCGCGGCACCT

Transcription levels were assessed daily from 6–15 doc. Quantitative PCR amplification of cDNA was carried out on a Mx3000P thermal cycler (Agilent Technologies, Santa Clara, CA, USA). The primers for gene amplification are listed in Table 1. Reaction mixtures contained 2.5 μl of sample DNA (100 ng/μl), 5 μl of SsoAdvanced™ Universal Probes Supermix (Bio-Rad) and 1.3 μl of nuclease-free water with primers and probes at a final concentration of 500 and 200 nM, respectively. Activation of polymerase was performed at 95 °C for 2 min, followed by 50 cycles of 95 °C for 15 s and 60 °C for 30 s. Each sample was run in triplicate and the complete experiment was performed in two separate biological replicates. The qPCR results were normalized against each of the two reference genes, namely glyceraldehyde-3-phosphate and actin. Average gene expression relative to the endogenous control for each sample was calculated using the 2^−ΔΔCq^ method.

### Statistical analysis

All values are expressed as means ± standard error of the mean (SEM). Significant differences among groups were compared using the Student’s t-test or one-way ANOVA with multiple comparisons. Differences were considered statistically significant at *P* < 0.05. All statistical analyses were conducted using GraphPad® Prism 8.2 (GraphPad Software, San Diego, CA).

## Results and discussion

### Morphology

**Fig. 1:**
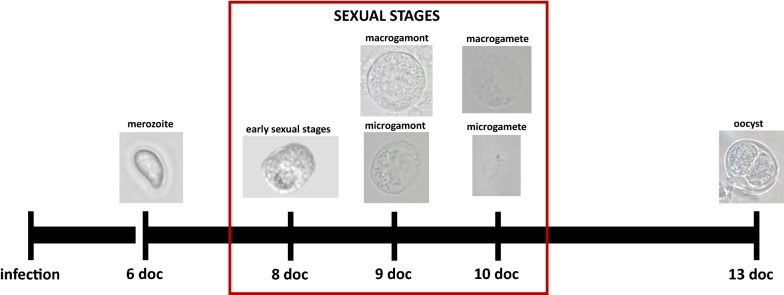
Time line of *C. suis* development *in vitro*. Early sexual stages could be found at 8 days of cultivation (doc) and gamonts from 9 doc onwards. Micro- and macrogametes could be detected from 10 doc onwards *in vitro*. Sporulated oocysts were found at 13 doc

Sporozoite-infected cell cultures are a suitable model for producing all stages of *C. suis in vitro.* Free merozoites were detected in appreciable numbers from day 6 doc. Sexual stages occured in appreciable numbers between 8–11 doc and were mainly found outside the host cell. Early sexual stages were first detected at 8 doc. First gamonts could be located from 9 doc onwards, whereas first macrogametes and motile microgametes could be found a day later (Fig. 1). We estimated that the ratio of gamonts:early sexual stages was about 1:2. The first oocysts appeared 11 doc until 13 doc.

The sexual development of coccidians, including *C. suis*, is not well characterized [8]. Although it is presumed that sexual stages are crucial in parasite development, little is known about their morphology, especially *in vitro* [6]. As the life-cycle of *C. suis* takes longer *in vitro* (about 11 days) than *in vivo* (5 days) [4], it is possible to collect samples of every stage of *C. suis* development, hence making sexual stages available for further research.

**Fig. 2:**
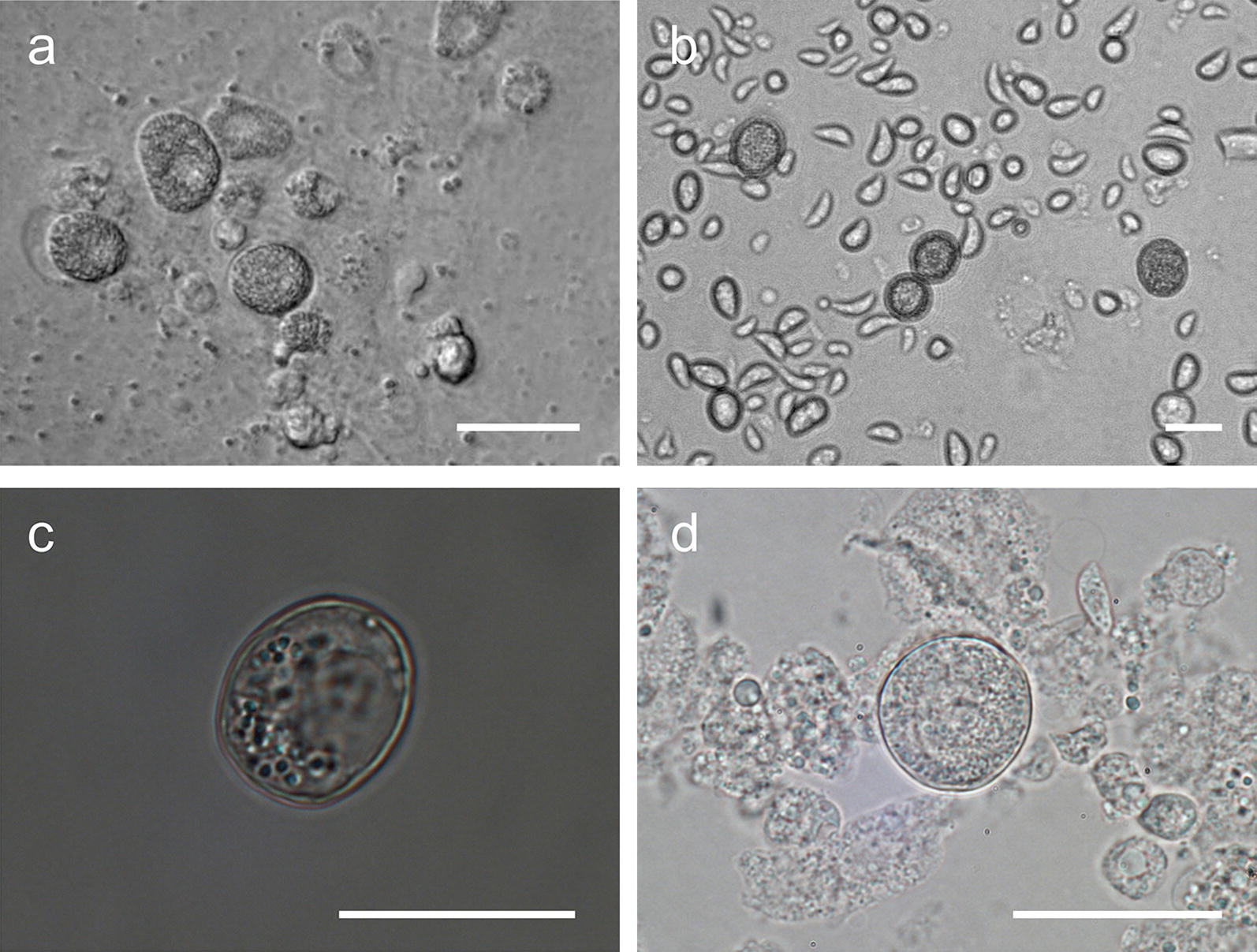
Light microscopy of different sexual stages of *C. suis in vitro* culture. **a** Early sexual stages, 7 days of cultivation (doc), differential interference contrast. **b** Micro- and macrogamont in close proximity to each other, 8 doc, differential interference contrast. **c** Microgamont, 8 doc. **d** Macrogamont, 8 doc. *Scale-bars*: 20 µm

Early sexual stages (immature gamonts) varied in form and size but their length was on average 15.6 ± 0.5 µm (*n* = 50) and their width 11.6 ± 0.4 µm (*n* = 50, Fig. 2a). Both micro- and macrogamonts were subspherical and had very similar diameter, however microgamonts were on average 16.8 ± 0.9 µm (*n* = 50), whereas macrogamonts were 16.6 ± 1.1 (*n* = 50) in average diameter (Table 2). In light microscopy, unstained microgamonts were recognized by their large vacuole and motile microgametes inside (Fig. 2b, c). Both micro- and macrogamonts were often found in close proximity to each other (Fig. 2b, d, Additional file 1), and the egress of microgametes from microgamonts could be observed during a 4-hour time frame. Each microgamont contained between 30–40 microgametes.

**Table 2:** Overview of mean, standard deviation, variance and range of *C. suis* sexual stages *in vitro* (*n* = 50)

	Early sexual stages		Microgamonts	Macrogamonts	Microgametes		Macrogamets
	Width	Length	Diameter	Diameter	Body	Tail	Diameter
Mean	11.6	15.6	16.8	16.6	3.5	11.2	12.1
Standard deviation	0.4	0.5	0.9	1.1	0.5	0.5	0.5
Variance	0.2	0.2	0.9	1.3	0.3	0.3	0.2
Minimum	11.0	15.0	15.4	15.1	3.0	10.8	11.5
Maximum	12.5	16.5	18.4	18.4	5.0	12.3	13.0

*Note*: All measurements are in micrometers

**Fig. 3:**
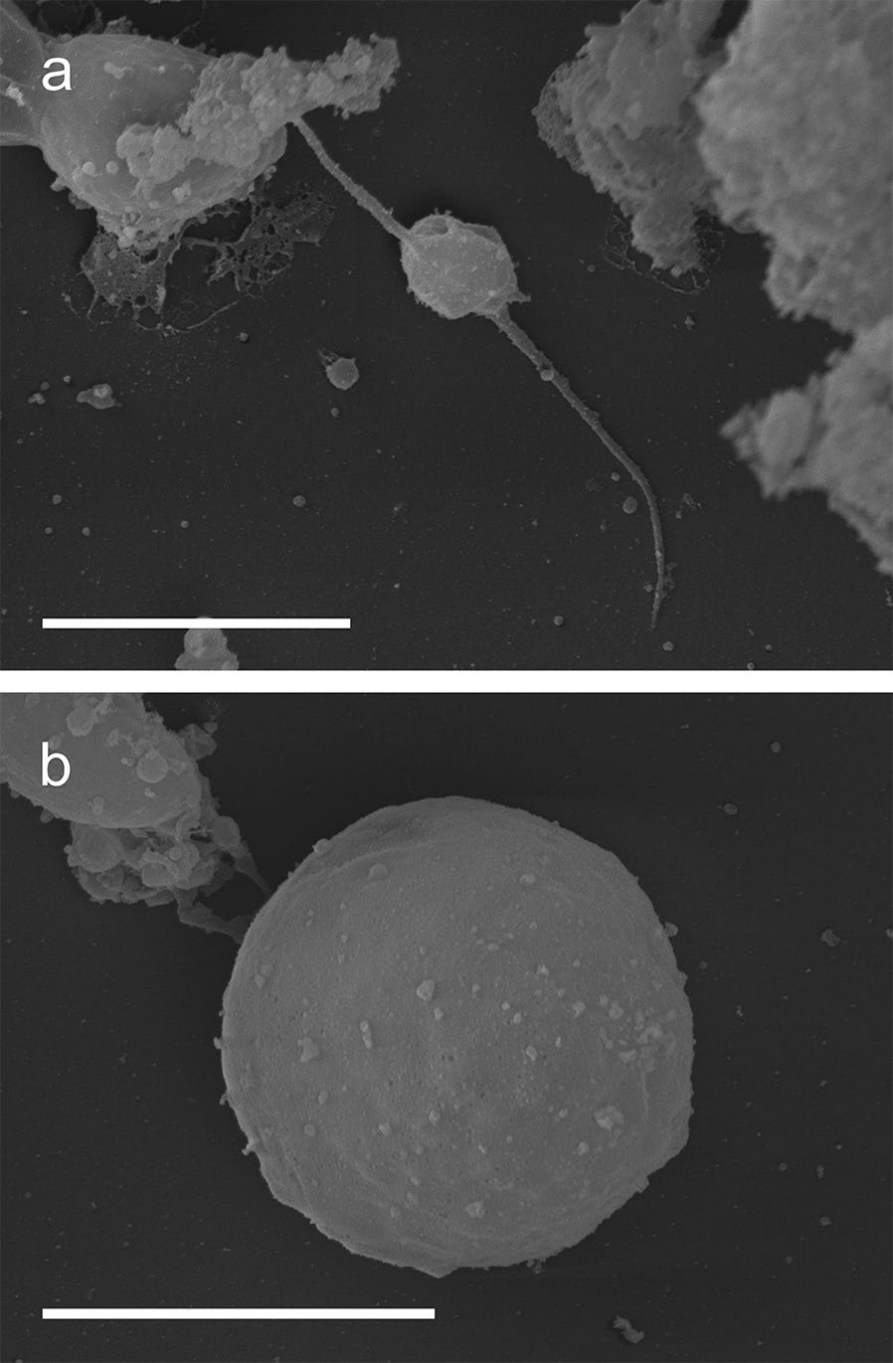
Scanning electron microscopy of adult sexual stages of *C. suis in vitro* culture. **a** Microgamete, 10 days of cultivation (doc). **b** Macrogamete from 10 doc. *Scale-bars*: 5 µm

Scanning electron microscopy observations showed that microgametes consisted of a small, spherical (3.5 ± 0.5 µm, *n* = 50) body with two opposing flagella, 11.2 ± 0.5 µm in length (*n* = 50, Fig. 3a), which enabled the quick movement of the microgamete in search for a macrogamete. Macrogametes on the other hand were immobile, spherical with a smooth surface and had a diameter of 12.1 ± 0.5 µm (*n* = 50; Fig. 3b, Table 2).

To our knowledge, this study provides the first detailed *in vitro* characterization of sexual stages of *C. suis*. Previous *in vivo* studies already described immature micro- and macrogamonts in tissue sections of the small intestine [5, 36]. Early sexual stages are described as ovoid to elongate and smaller in size than those *in vitro* (immature microgamonts: 11.8 × 8.4 µm; immature macrogamonts: 9.4 × 6.5 µm) while the shape and size of mature micro- and macrogamonts are comparable to those in the early sexual stages [6]. Other closely related coccidian species show similar morphology of the sexual stages [37, 38]; however, microgametes of *T. gondii* and *Eimeria* [17] have flagella on the posterior end, whereas in *C. suis* they are clearly positioned on opposite sides, which might also affect microgamete movement on the search for a macrogamete. With an *in vitro* system that allows for the collection and examination of mature sexual stages of *C. suis*, further studies on their properties and the fertilization process in Coccidia will be possible.

### Genes linked to sexual stages

Identification of genes linked to the sexual stages is crucial to understand the developmental biology and the fertilization process of organisms with sexual development, including apicomplexan protozoans. Here, we analyzed eight of the genes with highest upregulated transcripts in microgametes, macrogametes and oocysts based on previous studies in Apicomplexa [11, 12, 27, 29, 31, 39–42]. To identify genes or their orthologues related to the sexual development in *C. suis*, we used the ToxoDB parasite database and determined four genes related to sexual stages and four genes related to meiosis and cellular division (see Table 1).

For microgametes, the orthologues of dynein light chain 1 (*DLC1*) and the male gamete fusion factor (*HAP2*), *CsDLC1* (CSUI_000751) and *CsHAP2* (CSUI_000472), clustered with the respective genes from closely related coccidian parasites. Sequence analyses revealed identities greater than 60% with *T. gondii* and *Neospora caninum*, signifying the close relationship of *C. suis* with these two species [42], and more than 36% with *Eimeria necatrix* (Table 1).

Two of the most highly transcribed genes found in macrogametes code for proteins involved in the formation of the oocyst wall. *CsOWP1* (CSUI_006207) has more than 65% of identity with that of *T. gondii* (Table 1). Genome analysis of *C. suis* and *T. gondii* failed to identify orthologues for the GAM56 protein of *Eimeria* but revealed three low molecular weight hypothetical proteins possessing both a leader peptide and tyrosine-rich sequences. CSUI_001473 is an orthologue with 54% of homology with a gene coding for a tyrosine rich protein in *Toxoplasma* (TGME49_037080). Two of these three genes (TGME49_037080 and TGME49_087250) have peak expression levels in oocysts and the encoded proteins are incorporated as a part of the oocyst wall [40].

Among the genes with high expression in oocysts of *Toxoplasma* and *Cryptosporidium* [15, 40] we determined one coding for the meiotic recombination Rad51/Dmc1-like protein, and the orthologue in *C. suis*, CSUI_004539, had a 93% similarity to the gene of *Toxoplasma* (TGARI_272900) (Table 3).

**Table 3:** Classification of genes used in this study from *C. suis* and their comparison with other coccidian parasites

	*Cystoisospora suis*	*Toxoplasma gondii*		*Eimeria necatrix*		*Neospora caninum*	
	Accession no.	Accession no.	Identity (%)	Accession no.	Identity (%)	Accession no.	Identity (%)
Dynein light chain type *1* (*DLC1*)	CSUI_000751	TGARI_244900	71.70	ENH_00055140	36.49	NCLIV_019360	74.53
Male gamete fusion factor (*HAP2*)	CSUI_000472	TGARI_285940	63.80	ENH_00067440	37.5	NCLIV_014480	61.28
Oocyst Wall protein (*OWP1*)	CSUI_006207	TGARI_204420	65.87	ENH_00062180	23.45	NCLIV_020820	66.87
Tyrosine rich, “Eimeria gam-like”	CSUI_001473	TGARI_237080	54.97	ENH_00047090	22.52	NCLIV_050960	48.83
DNA repair proteinRad51/dmc1-like	CSUI_004539	TGARI_272900	93.18	ENH_00059490	81.98	NCLIV_059840	46.23
Nima*-*related protein kinase 1 *Nima1*	CSUI_004317	TGARI_292140A; TGARI_292140B	43.97	ENH_00060280	46.41	NCLIV_043340	42.44
Nima*-*related protein kinase 2 *Nima2*	CSUI_003099	TGARI_307640	86.62	ENH_00076430	65.94		
*Nima-*related protein kinase 4 *Nima4*	CSUI_000744	TGARI_244620	77.14	ENH_00003390	31.27	NCLIV_019170	83.46

*Notes*: Percentage values represent identities of *Toxoplasma*, *Eimeria* and *Neospora* genes with their corresponding *Cystoisospora* orthologs. All sequences are found in the genomic resource database ToxoDB (https://toxodb.org/toxo/). Alignment analyses were performed using the Clustal omega (https://www.ebi.ac.uk/Tools/msa/clustalo)

Protein kinases have a role not only for signalling, but also during the transition states of cells [43]. Nima (“never in mitosis-gene A”)-related kinases of *Plasmodium* and *Toxoplasma* are involved in post-fertilization processes and in the meiosis [32]. We found three orthologues for *Nima* genes, one of them specific for male gametocytes, *Nima1* (CSUI_004317) and two of them for female gametocytes, *Nima2* and *Nima4*, (CSUI_003099 and CSUI_000744) and all three showed more than 40% of homology with the respective genes of *Toxoplasma* (Table 3).

The expression profiles of one gene related to asexual stages (merozoites) of *C. suis* (CSUI_005805 [44]), the four genes related to sexual stages and the four genes related to cellular division and meiosis were examined at different time points of cultivation *in vitro* in extracellular and intracellular parasites.

**Fig. 4:**
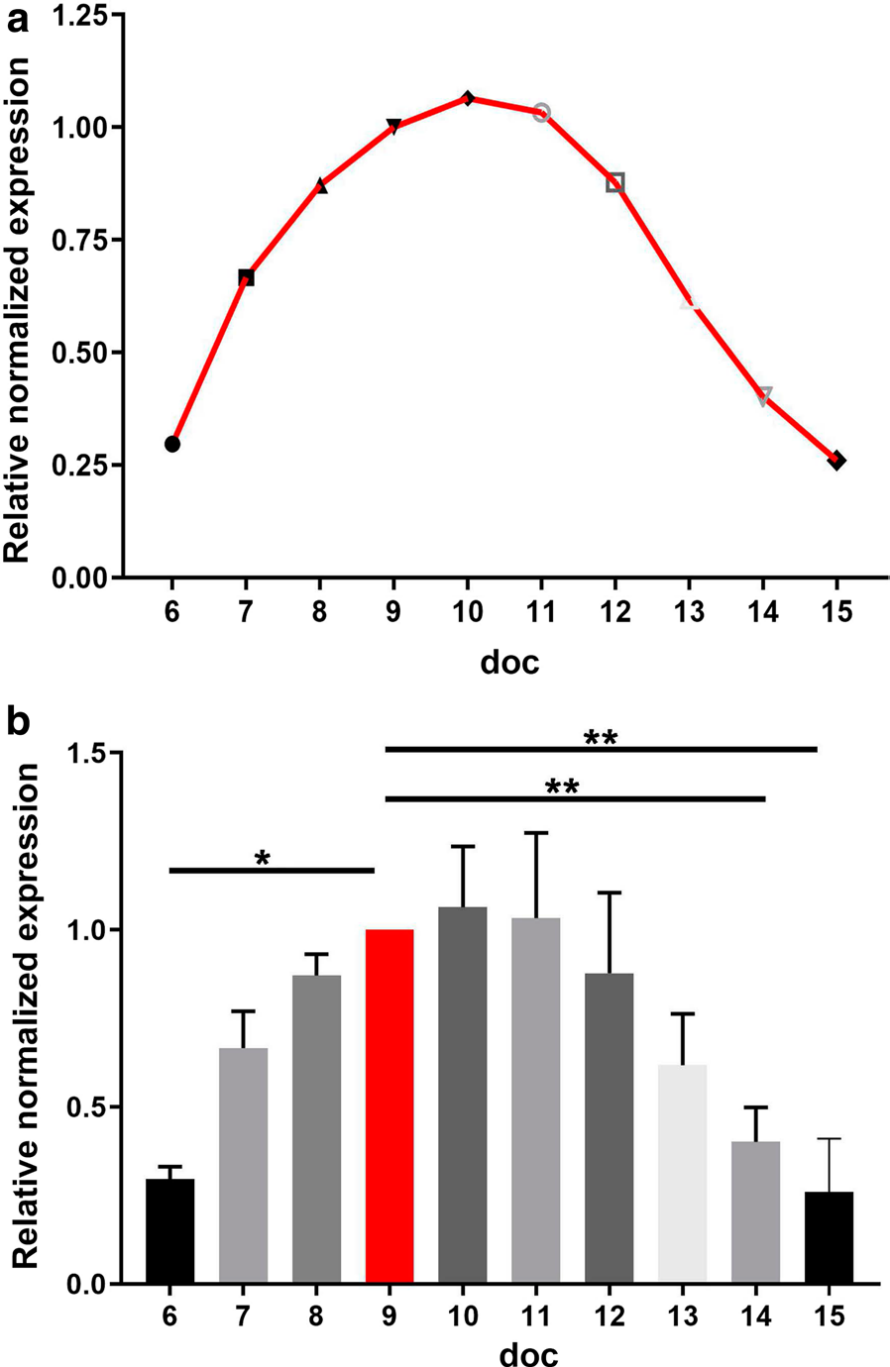
Relative mRNA expression levels of CSUI_005805 in intracellular parasites. Qualitative RT-PCR of intracellular parasites from 6–15 days of cultivation (doc). Values represent the mean ± standard error (SE) (*n* = 4). One-way ANOVA with multiple comparisons. **P < 0.01. For details on statistical tests see Additional file 2: Table S1

To evaluate the development of merozoites during cultivation and to test the suitability of the qPCR analysis for the detection of temporal changes in gene transcription, we included the uncharacterized merozoite-specific gene CSUI_005805. The level of the transcription for merozoites increased until it reached the maximum around 9–10 doc and then declined (Fig. 4). In previous studies, this gene showed a higher transcription in merozoites compared to sporozoites and in the present study, it showed higher levels during merozoite development compared to sexual development and oocyst formation. The transcriptional level increased throughout the entire merogony, indicating that this protein might be important for the establishment and/or growth of merozoites inside the host cell [44].

**Fig. 5:**
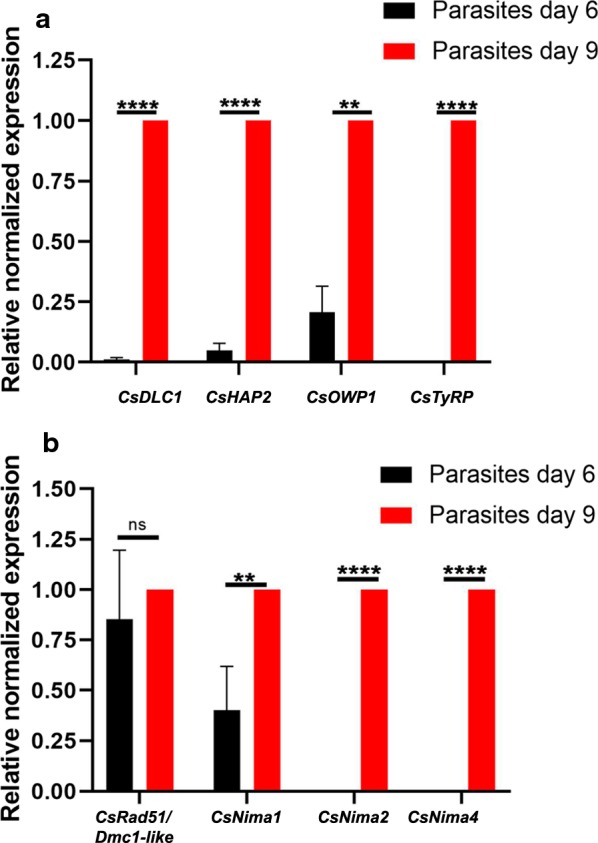
Relative mRNA expression levels of sexual related genes, cellular division and meiosis genes in extracellular parasites. Qualitative RT-PCR of intracellular parasites 6 to and 9 days of cultivation (doc). Values represent the mean ± standard error (SE) (*n* = 4). Unpaired Student’s t-test: ns: not significant (*P* > 0.05), **P < 0.01, ****P < 0.0001. For details on statistical tests see Additional file 2: Table S1

Transcript levels of genes related to sexual development in extracellular parasites were compared with 6 doc as a reference for merozoites released into the medium and 9–10 doc for extracellular gamonts/gametes. The transcription levels were 8–9-fold higher in gamonts/gametes compared to merozoites (Fig. 5a), which agrees with the high level of upregulation demonstrated in RNA-seq analysis of sexual stages of *Eimeria* spp. [11].

**Fig. 6:**
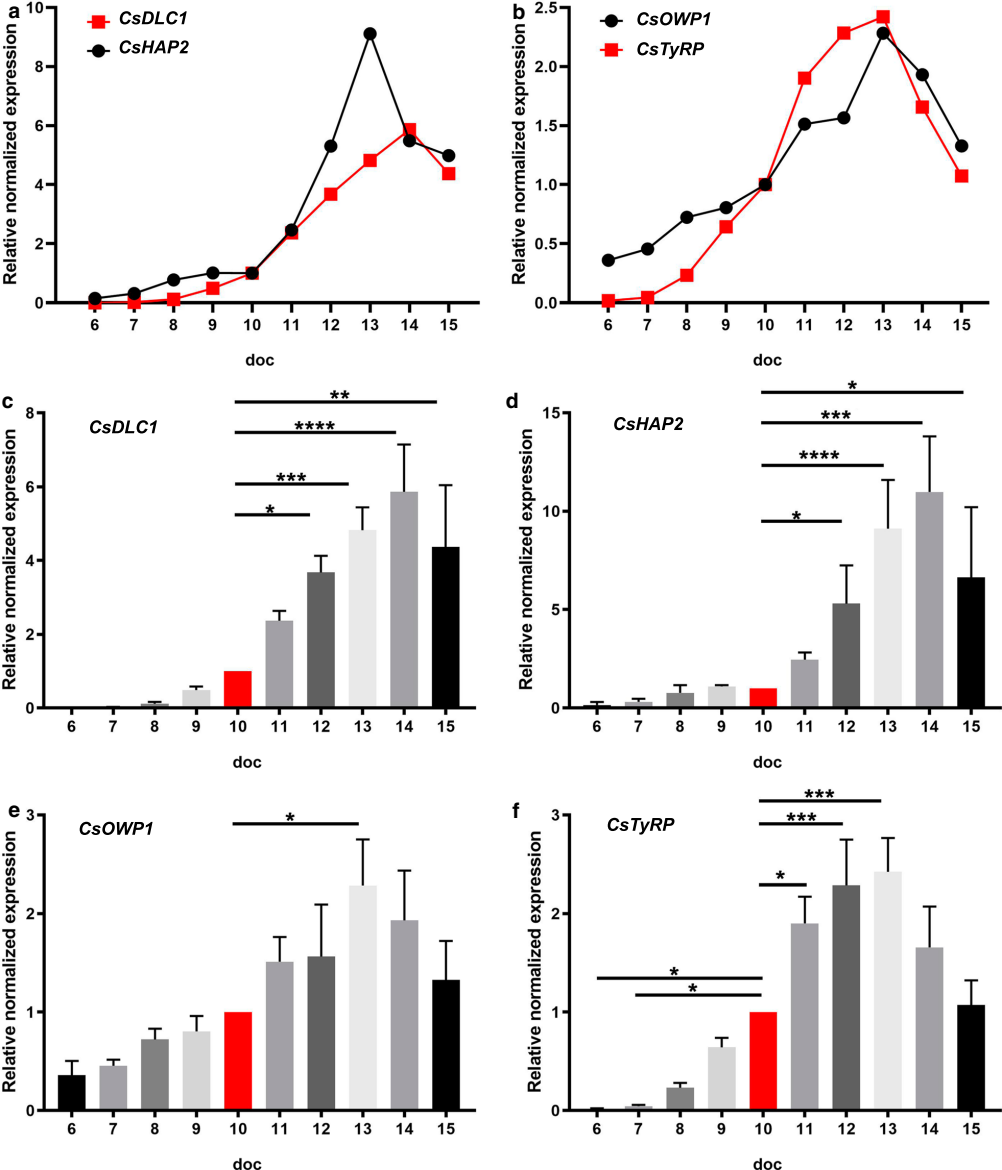
Relative mRNA expression levels of sexual related genes in intracellular parasites. qRT-PCR of intracellular parasites from day 6 to day 15 of cultivation. **a** Microgamete-related genes. **b** Macrogamete-related genes. **c***CsDLC1.***d***CsHAP2.***e***CsOWP1*. **f***CsTyRP* (CSUI_001473). Values represent the mean ± standard error (SE) from four independent experiments and are expressed as arbitrary absorbance units (*n* = 4). *P < 0.05, **P < 0.01***, *P* < 0.001, ****P < 0.0001. For details on statistical tests (one-way ANOVA with multiple comparisons) see Additional file 2: Table S1

Transcription levels of intracellular parasites were compared daily at 6–15 doc. As a reference point, we used day 10 because on that day first mature gametes were seen *in vitro*. The microgamete-related genes transcripts reach peaks on 13–14 doc and the increase was 10–90-fold higher compared to merozoites at 6 doc (Fig. 6) which is in agreement with results for RNA seq comparing merozoites from third-generation merozoites and gamonts of *Eimeria* [11]. The levels for *DLC1* and *HAP2* (Fig. 6a, c, d) transcripts on 13 and 14 doc were 30-fold and 60-fold higher, respectively, compared to day 6 with no detectable expression. Dynein proteins are part of the flagellum of microgametes and form part of the microtubule motor [45]. They are also involved in mitosis and meiosis, and are the major constituents of mitotic spindles, which are used to pull eukaryotic chromosomes apart [46]. As judged from the gene upregulation, these processes take place in *C. suis in vitro* between 9–15 doc. However, before that time point no transcription of DLC1 could be measured and the expression of DLC1 in Apicomplexa seems to be restricted to microgametes as described earlier [11].

In addition, we observed an upregulation of the gene expression of the fusogen *HAP2* during 9–13 doc followed by a decrease (Fig. 6a, d). Its expression is also restricted to male gametes and its function in gamete fusion and during fertilization is extensively described for plants and unicellular eukaryotes [26, 47]. The results in *C. suis* correlate with previous reports on *Eimeria* and *Toxoplasma*. HAP2 is found in microgametes and unsporulated oocysts but not in sporulated oocysts or sporozoites [26, 27, 40, 48, 49]. Moreover, transcription of *HAP2* was increased during the enteric development of *T. gondii* in the intestine of cats. *HAP2* knockout parasites failed to fertilize and produce oocysts *in vivo*, and this supports the hypothesis that interfering with the fertilization process can be utilized in a transmission-blocking vaccine [29]. As for *DLC1*, expression seems to be restricted to microgametes.

Oocyst wall formation is a hallmark of coccidian development, and *OWP* and *GAM*-encoded proteins have previously been characterized in *Eimeria* and *Toxoplasma* as well as *Cryptosporidium* as constituents of the oocyst wall [29, 50, 51].

The GAM proteins EmGam56 and EmGam82 were identified as antigens that conferred protection against different species of chicken *Eimeria* due to their conserved nature. A subunit vaccine for immunization was previously developed from *E. maxima* gamont proteins and commercialized for the prevention of coccidiosis [9, 52].

No homologues for GAM genes were found in the *C. suis* genome or in the *Toxoplasma* database. However, oocysts of *C. suis* display the characteristic autofluorescence similar to other coccidia [17, 53] which is likely due to the dityrosine bonds formed between tyrosine-rich proteins present in the oocyst wall [11, 51]. A search in the proteome of *Toxoplasma* for predicted proteins with sequences rich in tyrosine identified five hypothetical proteins, and three of them presented highest levels of expression in the oocyst wall proteomic fraction [31] and were upregulated in oocyst transcriptomes compared to tachyzoites and bradyzoites [40]. We identified and analyzed an orthologue of one of them, TyRP. Gene transcripts of both proteins presumably involved in oocyst wall formation, *OWP1* and *TyRP*, were upregulated until 13 doc and then steeply declined (Fig. 6b, e, f). While *TyRP* transcription could only be detected from 8 doc (Fig. Fig. 6b, f), low transcription levels for *OWP1* were already found from 6 doc (Fig. 6b, e). indicating that merozoites present at this time point are probably already committed to a further development into macrogamonts, as described for *E. tenella* [17, 27].

**Fig. 7:**
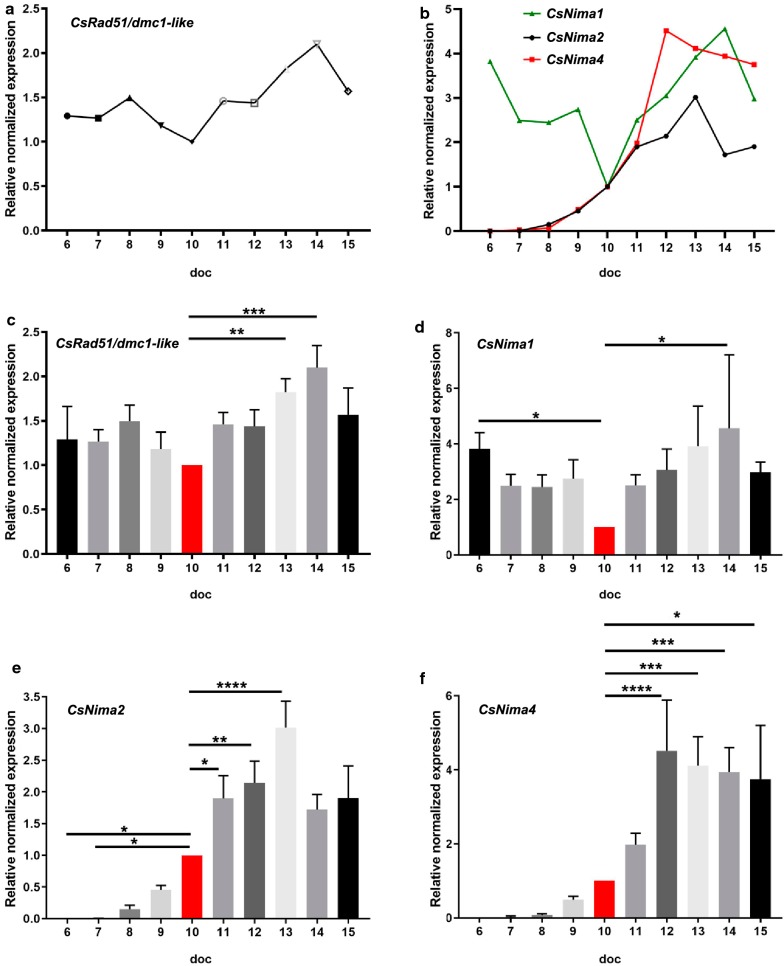
Relative mRNA expression levels of related to cellular division and meiosis genes in intracellular parasites. qRT-PCR of intracellular parasites from 6 to 15 days of cultivation (doc). **a***CsRad51-*related gene. **b***Nima-Nek-*related kinase genes. **c***CsRad51*. **d***CsNima1*. **e***CsNima2*. **f***CsNima4*. Values represent the mean ± standard error (SE) from four independent experiments and are expressed as arbitrary absorbance units (*n* = 4). *P < 0.05, **P < 0.01***, *P* < 0.001, ****P < 0.0001. For details on statistical tests (one-way ANOVA with multiple comparisons) see Additional file 2: Table S1

In extracellular parasites, the RNA transcription of *Rad51/Dmc1-like* showed similar levels of transcription in parasites from 6 and 9–10 doc (Fig. 5b). In intracellular stages of *C. suis*, the RNA transcription levels of *Rad51/Dmc1-like* were rather constant from 6–12 doc except for an increase 13–14 doc (Fig. 7a, c) which probably corresponds to meiosis during the formation of the oocyst, since the *RAD51/Dmc1-like* gene codes for a protein of the Rad51 family which assists in repair of DNA double strand breaks during mitosis, while the two recombinases, Rad51 and Dmc1, facilitate the recombination between homologous chromosomes during meiosis [54].

Nima-related kinases (Nek or NRK) represent a conserved family of serine/threonine kinases implicated in the regulation of distinct cellular events [47]. Neks have important roles in the maintenance of centrosome function and structure, mitotic microtubule organization, and the regulation of axonemal microtubule in cilia and flagella. Nima1 is an orthologue of Nek2 in humans which is involved in the maintenance of centrosome structures and mitotic microtubule organization, thus playing a role during mitosis. In *Toxoplasma*, Nima1 is essential for centrosome splitting, proper formation of daughter cell budding and faithful segregation of genetic material. A point mutation in a conserved portion of the gene causes a severe mitotic defect [48]. In *Plasmodium*, Nima1 is found in replicative forms of the parasites, in asexual and sexual stages, with a role in mitosis, and specifically in microgametes. In extracellular parasites, Nima1 showed almost double the expression levels in gamonts compared to parasites at 6 doc, and Nima2 and Nima4 were almost exclusively transcribed in gamonts/gametes (Fig. 5b). The mRNA levels in intracellular parasites found for Nima1 were significantly higher on 6 and 14 doc compared to 10 doc (Fig. 7 b, d). Our results correlate with the presence of Nima1 in asexual stages and sexual stages [49]. Due to the simultaneous presence of both in cell culture an upregulation in sexual stages could not be observed. By contrast, Nima2 and Nima4 were practically absent before 9 doc when the first mature gamonts occurred and peaked at 13 and 14 doc, respectively (Fig. 7b, e, f). In *Plasmodium*, Nima2 and Nima4 are only found in female gametocytes and the two encoded proteins are necessary for completion of the sexual cycle. In Nek2 knockout parasites premeiotic DNA replication is dysregulated and the parasites do not develop ookinetes, suggesting that the principle role of Nek2 is during DNA replication preceding the meiosis Nek 4 does not appear to be required for gametocytogenesis but is essential for premeiotic DNA replication in the zygote, consistent with the cell cycle related function [55]. As these two *Nima* genes were expressed in parallel with the occurrence of gamonts and unsporulated oocysts *in vitro* we assume that the encoded proteins have similar roles during the development of *C. suis*.

## Conclusions

Although sexual stages of Coccidia have previously been addressed for intervention in *T. gondii* infections, the lack of models for detailed studies on the involved stages *in vitro* has been highly prohibitive for more detailed research. In the present study, we could demonstrate mature gamonts, gametes and oocysts of *C. suis in vitro* in a defined time frame as well as a correlation of size and form of stages *in vitro* with those found *in vivo*. We also identified genes linked to the developmental and cell cycle progression of *C. suis in vitro*. We defined the demonstration of sexual stages *in vitro*, their time-limited occurrence and the gene expression of stage-specific genes. It was previously demonstrated in other coccidians, *Eimeria* and *Toxoplasma*, that interfering with fertilisation can block transmission of this parasite, providing a novel tool for intervention strategies and a hint at a potential developmental bottleneck in the life-cycle of *C. suis.*

## Supplementary information

**Additional file 1.** Video of free microgametes, a macrogamont containing motile microgametes and a macrogamete in close proximity to each other, in real time.

**Additional file 2: Table S1.** Reporting of significant results from statistical analyses from Figs. 4, 5, 6, 7.

## Data Availability

All data and materials of the experiments described here are included in this published article and its additional files.

## Abbreviations

BLAST: basic local alignment search tool
DLC1: dynein light chain type 1 protein
GAM: sexual (macrogamete/macrogametocyte) stage proteins (wall-forming proteins, wfp)
HAP2: male gamete fusion factor hapless-2
IPEC: intestinal porcine epithelial cells
Nima: “never in mitosis-A”-related kinases
Nek/NRK: Nima-related kinases
OWP1: oocyst wall protein 1
qRT-PCR: quantitative real time polymerase chain reaction
Rad51: DNA repair protein 51
TyRP: tyrosine rich protein
