# Supplementary material for: Characterization of Cystoisospora suis sexual stages in vitro
Source: Parasit Vectors. 2020 Mar 18;13:143. doi: 10.1186/s13071-020-04014-4 (PMC7079422; doi:10.1186/s13071-020-04014-4)
Supplement: Supplementary file 2 — Additional file 2: Table S1. Reporting of significant results from statistical analyses from Figs. 4, 5, 6, 7. [file 13071_2020_4014_MOESM2_ESM.docx]

| Test Details *CSUI_005805* | Significant | Adjusted *P* Value | *df* |
| --- | --- | --- | --- |
| 10 vs. 6 | * | 0.0106 | 41 |
| 10 vs. 7 | ns | 0.3210 | 41 |
| 10 vs. 8 | ns | 0.9479 | 41 |
| 10 vs. 9 | ns | 0.9995 | 41 |
| 10 vs. 11 | ns | 0.9998 | 41 |
| 10 vs. 12 | ns | 0.9363 | 41 |
| 10 vs. 13 | ns | 0.1480 | 41 |
| 10 vs. 14 | ** | 0.0084 | 41 |
| 10 vs. 15 | ** | 0.0022 | 41 |

**Additional file 2: Table S1.** Reporting of significant results from statistical analyses from Figs. 4–7.

| Test Details | Significant | t ratio | *df* | *P* Value |
| --- | --- | --- | --- | --- |
| *CsDLC1* | **** | 109.5 | 6.000 | <0.000001 |
| *CsHAP2* | **** | 33.19 | 6.000 | <0.000001 |
| *CsOWP1* | ** | 7.355 | 6.000 | 0.000323 |
| *CsTyRP* | **** | 807.2 | 6.000 | <0.000001 |
| *CsRad51/dmc1-*like | ns | 0.4299 | 4.000 | 0.689427 |
| *CsNima1* | ** | 5.848 | 4.000 | 0.008513 |
| *CSNima2* | **** | 9999 | 4.000 | <0.000001 |
| *CsNima4* | **** | 4802 | 4.000 | <0.000001 |

| Test Details *CsDLC1* | Significant | Adjusted *P* Value | *df* |
| --- | --- | --- | --- |
| 10 vs. 6 | ns | 0.8400 | 43 |
| 10 vs. 7 | ns | 0.8531 | 43 |
| 10 vs. 8 | ns | 0.8759 | 43 |
| 10 vs. 9 | ns | 0.9885 | 43 |
| 10 vs. 11 | ns | 0.4509 | 43 |
| 10 vs. 12 | * | 0.0105 | 43 |
| 10 vs. 13 | *** | 0.0001 | 43 |
| 10 vs. 14 | **** | <0.0001 | 43 |
| 10 vs. 15 | ** | 0.0019 | 43 |

| Test Details *CsHAP2* | Significant | Adjusted *P* Value | *df* |
| --- | --- | --- | --- |
| 10 vs. 6 | ns | 0.9994 | 37 |
| 10 vs. 7 | ns | 0.9994 | 37 |
| 10 vs. 8 | ns | 0.9998 | 37 |
| 10 vs. 9 | ns | >0.9999 | 37 |
| 10 vs. 11 | ns | 0.9375 | 37 |
| 10 vs. 12 | * | 0.0390 | 37 |
| 10 vs. 13 | **** | <0.0001 | 37 |
| 10 vs. 14 | *** | 0.0002 | 37 |
| 10 vs. 15 | * | 0.0187 | 37 |

| Test Details *CsOWP1* | Significant | Adjusted *P* Value | *df* |
| --- | --- | --- | --- |
| 10 vs. 6 | ns | 0.6895 | 40 |
| 10 vs. 7 | ns | 0.8446 | 40 |
| 10 vs. 8 | ns | 0.9980 | 40 |
| 10 vs. 9 | ns | 0.9995 | 40 |
| 10 vs. 11 | ns | 0.8846 | 40 |
| 10 vs. 12 | ns | 0.7444 | 40 |
| 10 vs. 13 | * | 0.0134 | 40 |
| 10 vs. 14 | ns | 0.1480 | 40 |
| 10 vs. 15 | ns | 0.9967 | 40 |

| Test Details *CsTyRP* | Significant | Adjusted *P* Value | *df* |
| --- | --- | --- | --- |
| 10 vs. 6 | * | 0.0331 | 44 |
| 10 vs. 7 | * | 0.0407 | 44 |
| 10 vs. 8 | ns | 0.1051 | 44 |
| 10 vs. 9 | ns | 0.7509 | 44 |
| 10 vs. 11 | * | 0.0360 | 44 |
| 10 vs. 12 | *** | 0.0010 | 44 |
| 10 vs. 13 | *** | 0.0002 | 44 |
| 10 vs. 14 | ns | 0.2264 | 44 |
| 10 vs. 15 | ns | 0.9997 | 44 |

| Test Details *Rad51/dmc1-*like | Significant | Adjusted *P* Value | *df* |
| --- | --- | --- | --- |
| 10 vs. 6 | ns | 0.9130 | 41 |
| 10 vs. 7 | ns | 0.9076 | 41 |
| 10 vs. 8 | ns | 0.3057 | 41 |
| 10 vs. 9 | ns | 0.9698 | 41 |
| 10 vs. 11 | ns | 0.3057 | 41 |
| 10 vs. 12 | ns | 0.3633 | 41 |
| 10 vs. 13 | ** | 0.0071 | 41 |
| 10 vs. 14 | *** | 0.0002 | 41 |
| 10 vs. 15 | ns | 0.1734 | 41 |

| Test Details *CsNima1* | Significant | Adjusted *P* Value | *df* |
| --- | --- | --- | --- |
| 10 vs. 6 | * | 0.0403 | 33 |
| 10 vs. 7 | ns | 0.6069 | 33 |
| 10 vs. 8 | ns | 0.6413 | 33 |
| 10 vs. 9 | ns | 0.5476 | 33 |
| 10 vs. 11 | ns | 0.5081 | 33 |
| 10 vs. 12 | ns | 0.1724 | 33 |
| 10 vs. 13 | ns | 0.0658 | 33 |
| 10 vs. 14 | * | 0.0142 | 33 |
| 10 vs. 15 | ns | 0.3936 | 33 |

| Test Details *CsNima2* | Significant | Adjusted *P* Value | *df* |
| --- | --- | --- | --- |
| 10 vs. 6 | * | 0.0324 | 43 |
| 10 vs. 7 | * | 0.0335 | 43 |
| 10 vs. 8 | ns | 0.0612 | 43 |
| 10 vs. 9 | ns | 0.3269 | 43 |
| 10 vs. 11 | * | 0.0416 | 43 |
| 10 vs. 12 | ** | 0.0047 | 43 |
| 10 vs. 13 | **** | <0.0001 | 43 |
| 10 vs. 14 | ns | 0.1602 | 43 |
| 10 vs. 15 | ns | 0.0666 | 43 |

| Test Details *CsNima4* | Significant | Adjusted *P* Value | *df* |
| --- | --- | --- | --- |
| 10 vs. 6 | ns | 0.5954 | 38 |
| 10 vs. 7 | ns | 0.6223 | 38 |
| 10 vs. 8 | ns | 0.5906 | 38 |
| 10 vs. 9 | ns | 0.9488 | 38 |
| 10 vs. 11 | ns | 0.5322 | 38 |
| 10 vs. 12 | **** | <0.0001 | 38 |
| 10 vs. 13 | *** | 0.0001 | 38 |
| 10 vs. 14 | *** | 0.0003 | 38 |
| 10 vs. 15 | * | 0.0145 | 38 |
